# Supplementary material for: The dicarbon bonding puzzle viewed with photoelectron imaging
Source: Nat Commun. 2019 Nov 15;10:5199. doi: 10.1038/s41467-019-13039-y (PMC6858380; doi:10.1038/s41467-019-13039-y)
Supplement: Supplementary file 1 — Supplementary Information [file 41467_2019_13039_MOESM1_ESM.pdf]

Supplementary Information

The dicarbon bonding puzzle viewed with photoelectron imaging

Laws et al.

SUPPLEMENTARY DISCUSSION

Rotational band models for the $C_2(X^1\Sigma_g^+) \leftarrow C_2^-(X^2\Sigma_g^+)$ and $C_2(a^3\Pi_u) \leftarrow C_2^-(X^2\Sigma_g^+)$ electronic transitions have been constructed using methods similar to those developed by Buckingham *et al.* [1]. The anion $^2\Sigma_g^+$ and ground neutral $^1\Sigma_g^+$ states belong to Hund's case (b), where there is negligible spin-orbit coupling. The positions of the rotational lines of the anion $^2\Sigma_g^+$ state are given by the formula of Mulliken [2],

$$F_{1,2} = BN(N+1) \pm \frac{1}{2}\gamma N, \tag{1}$$

where F_1 and F_2 are the doublet fine structure components, B is the rotational constant, N is the total rotational (minus spin) angular momentum quantum number, and γ is the spin-rotation splitting constant ($= 0.001274\,\mathrm{cm}^{-1}$ [3]). For the neutral ground state, where there is no spin splitting, the levels are simply given by, $F = BN(N+1)$.

The excited $^3\Pi_u$ state of the neutral belongs to Hund's case (a) as it experiences strong spin-orbit (LS) coupling. For triplet Π states, the rotational levels are given by [4],

$$F_1 = B\left[J(J+1) - \sqrt{Z_1} - 2Z_2\right], \tag{2}$$
$$F_2 = B\left[J(J+1) + 4Z_2\right], \tag{3}$$
$$F_3 = B\left[J(J+1) + \sqrt{Z_1} - 2Z_2\right], \tag{4}$$

with the values Z_1 and Z_2 defined as,

$$Z_1 = Y(Y-4) + \frac{4}{3} + 4J(J+1), \tag{5}$$
$$Z_2 = \frac{1}{3Z_1}\left[Y(Y-1) - \frac{4}{9} - 2J(J+1)\right], \tag{6}$$

where B is the rotational constant of the molecule, Y is the ratio of the spin-orbit and rotational constants $Y = A_{\mathrm{SO}}/B$, and J is the total angular momentum quantum number.

The intensity of each rotational transition may be calculated using the method of Ref. [1], which employs Wigner $3j$-symbols to describe the angular momentum couplings involved in the transitions. For the ground state $C_2(X^1\Sigma_g^+) \leftarrow C_2^-(X^2\Sigma_g^+)$ photodetachment, the intensity Q-factor for a case-(b) to case-(b) transition is,

$$Q = (2N'+1)\begin{pmatrix} N' & j_t & N'' \\ -\Lambda' & \Delta\Lambda & \Lambda'' \end{pmatrix}^2, \tag{7}$$

where j_t is a variable ranging from $|\ell - 1|$ to ℓ, and Λ is the axial component of the orbital angular momentum. For transitions to the ground state of C_2, $\Lambda' = \Lambda'' = \Delta\Lambda = 0$.

Similarly, the rotational transition intensities involved in the excited state $C_2(a^3\Pi_u) \leftarrow C_2^-(X^2\Sigma_g^+)$ photodetachment may be calculated using the Q-factor for a case-(b) to case-(a) transition,

$$Q = \frac{2J'+1}{2S'+1}\sum_{\chi=j_t-1/2}^{j_t+1/2}(2\chi+1)\begin{pmatrix} j_t & S' & \chi \\ \Delta\Lambda & \Sigma' & \Lambda''-\Omega' \end{pmatrix}^2 \begin{pmatrix} J' & \chi & N'' \\ -\Omega' & \Omega'-\Lambda'' & \Lambda'' \end{pmatrix}^2, \tag{8}$$

where S' is the spin of the neutral, Σ' is the axial component of the spin, and Ω' is the projection of the total electronic angular momentum of the molecular state onto the molecular axis. For detachment yielding $C_2(a^3\Pi_u)$, $S' = 1,\ \Lambda' = 1,\ \Lambda'' = 0,\ \Delta\Lambda = 1,$ and $\Omega' = \Lambda' + \Sigma'$.

The rotational model defined above was fitted to the experimental photoelectron spectrum, as shown in Fig. **??**. Fitting the model yields an anion rotational temperature of T=197(2) K, while the Gaussian full-width half-maximum (FWHM) of each transition gives an energy resolution of $\Gamma_{X^1\Sigma} = 17(1)$ cm^{-1} and $\Gamma_{a^3\Pi} = 19(1)$ cm^{-1}, which are consistent with expectations for the spectrometer at kinetic energies of $\epsilon \sim 0.5$ eV.

The above rotational model is fitted to the experimental photoelectron spectrum in Fig. 4. This provides an accurate determination of the band head origin, allowing for precise determination of spectroscopic constants, as presented in Supplementary Table I.

SUPPLEMENTARY TABLES

Supplementary Table I: Molecular constants (cm^{-1}) of C_2 and C_2^-.

		HR-PEI	Previous Work
C_2^- $X^2\Sigma_g^+$	B	1.746(1)	1.7468(5)[a]
	ω_e	1782(2)	1781.189(18)[a]
C_2 $X^1\Sigma_g^+$	EA	26,396(3)	26,360(50)[b]
	B	1.820(1)	1.8198(4)[c]
C_2 $a^3\Pi_u$	T_e	718(1)	718.318(1)[d]
	A_{SO}	$-14.6(6)$	$-15.272(3)$[d]
	B	1.635(4)	1.6324(6)[d]

[a] from infrared study of Rehfuss *et al.* [5]
[b] from photoelectron spectroscopy of Lineberger *et al.* [6]
[c] from infrared study of Davis *et al.* [7]
[d] from flame-emission spectroscopy of Amiot *et al.* [8]

SUPPLEMENTARY REFERENCES

[1] A. D. Buckingham, B. J. Orr, and J. M. Sichel, Phil. Trans. R. Soc. A **268**, 147 (1970).
[2] R. S. Mulliken, Rev. Mod. Phys. **2**, 60 (1930).
[3] K. Huber and G. Herzberg, *Molecular spectra and molecular structure*, Vol. IV (Van Nostrand Reinhold Company, 1979).
[4] G. Herzberg, *Molecular Spectra and Molecular Structure - Spectra of Diatomic Molecules*, Vol. I (Van Nostrand Reinhold Company, 1950) pp. 110–140, 168–212.
[5] B. D. Rehfuss, D. Liu, B. M. Dinelli, M. Jagod, W. C. Ho, M. W. Crofton, and T. Oka, J. Chem. Phys. **89**, 129 (1988).
[6] K. M. Ervin and W. C. Lineberger, J. Phys. Chem. **95**, 1167 (1991).
[7] S. P. Davis, M. C. Abrams, J. G. Phillips, and M. L. P. Rao, J. Opt. Soc. Am. B **5**, 2280 (1988).
[8] C. Amiot, J. Chauville, and J.-P. Maillard, J. Mol. Spectrosc. **75**, 19 (1979).
